# Supplementary material for: Fluctuating Environments Maintain Genetic Diversity through Neutral Fitness Effects and Balancing Selection
Source: Mol Biol Evol. 2021 Jun 16;38(10):4362–75. doi: 10.1093/molbev/msab173 (PMC8476146; doi:10.1093/molbev/msab173)
Supplement: msab173_Supplementary_Data [file msab173_supplementary_data.zip › Abdul-Rahman et al Supplementary Figures and Tables.pdf]

SUPPLEMENTARY FIGURES

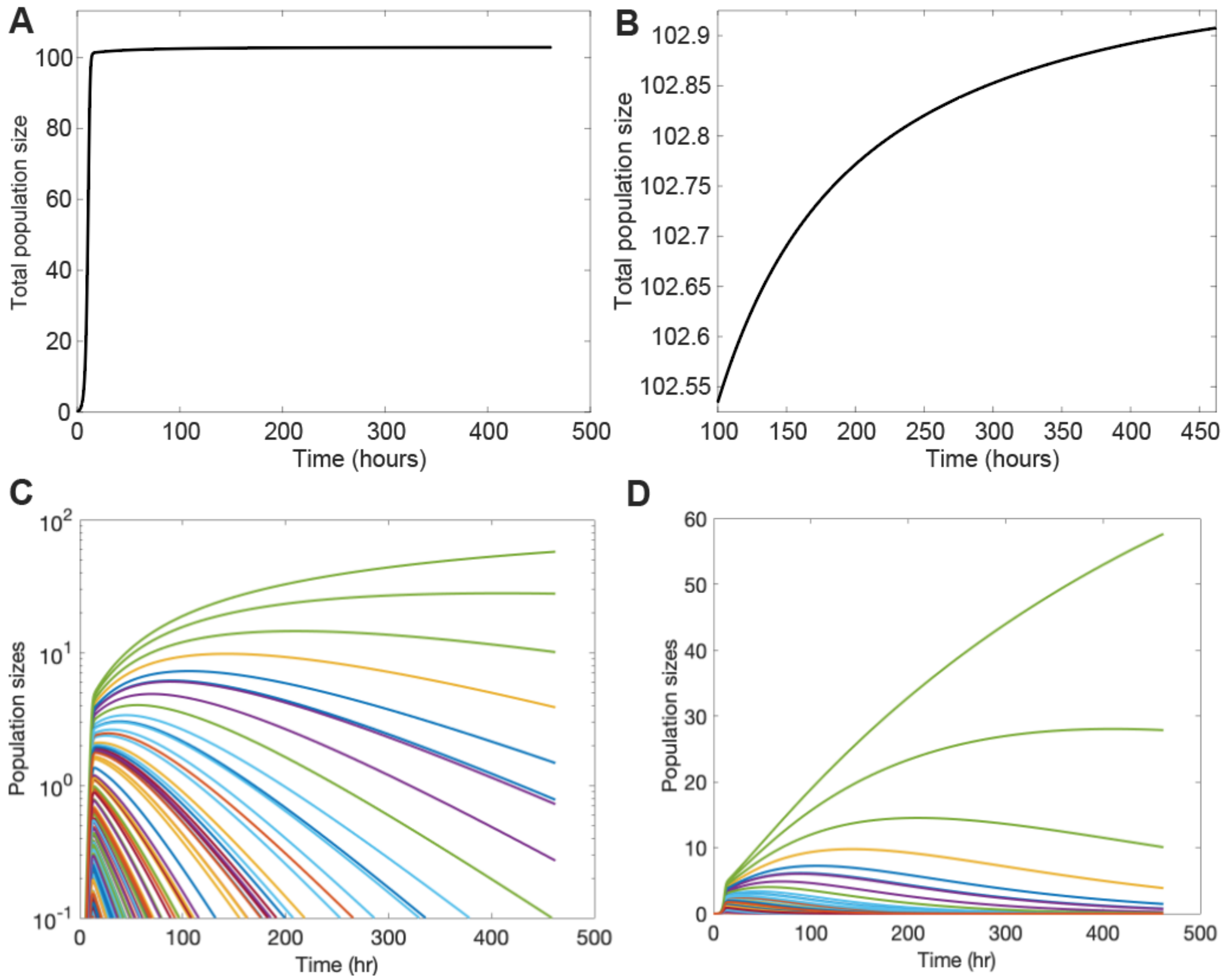

Supplemental figure 1. Modelling the growth of four thousand genotypes in the chemostat. (A) In the presence of thousands of genotypes the chemostat attains a quasi-steady state. **(B)** The total population size undergoes non-zero, but negligible, changes as selection acts on the population. **(C)** Individual genotype population sizes undergo large changes in frequency despite the relative invariance of total population size. (**D**) Dynamics of the top ten lineages.

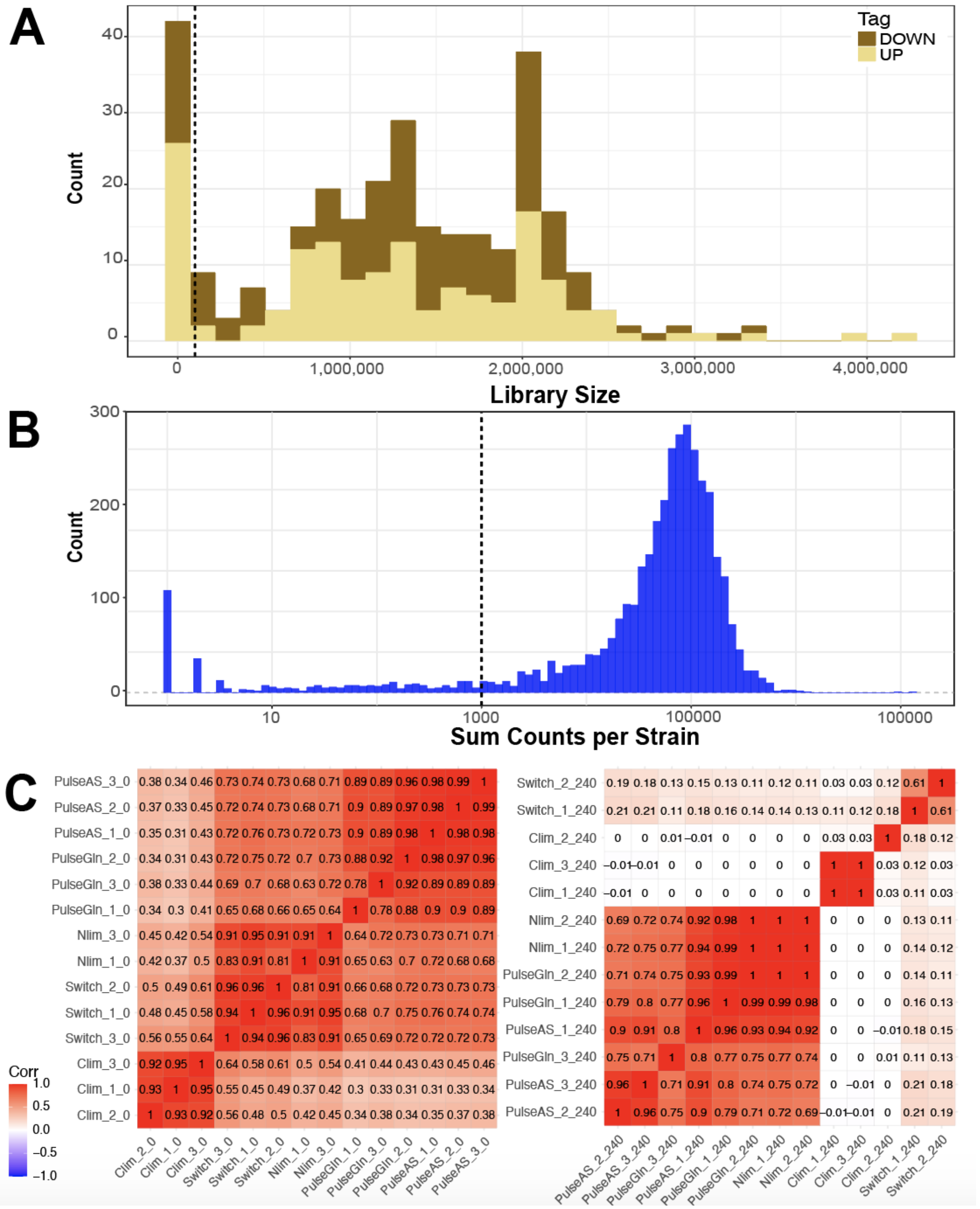

Supplemental figure 2. Barseq library quality control. (**A**) The complete distribution of library sizes before filtering is shown. The dashed line indicates libraries with less than 100,000 reads that were excluded from subsequent analysis. (**B**) The distribution of aggregate counts per genotype across all libraries. The dashed line indicates genotypes with less than 1,000 aggregate reads that were excluded from subsequent analyses. (**C**) Pairwise Pearson correlation coefficients between all samples at the first (t = 0) (**left panel)** and final time point (t = 240) (**right panel**). Replicates with correlation coefficients less than 0.6 were removed before proceeding with analysis.

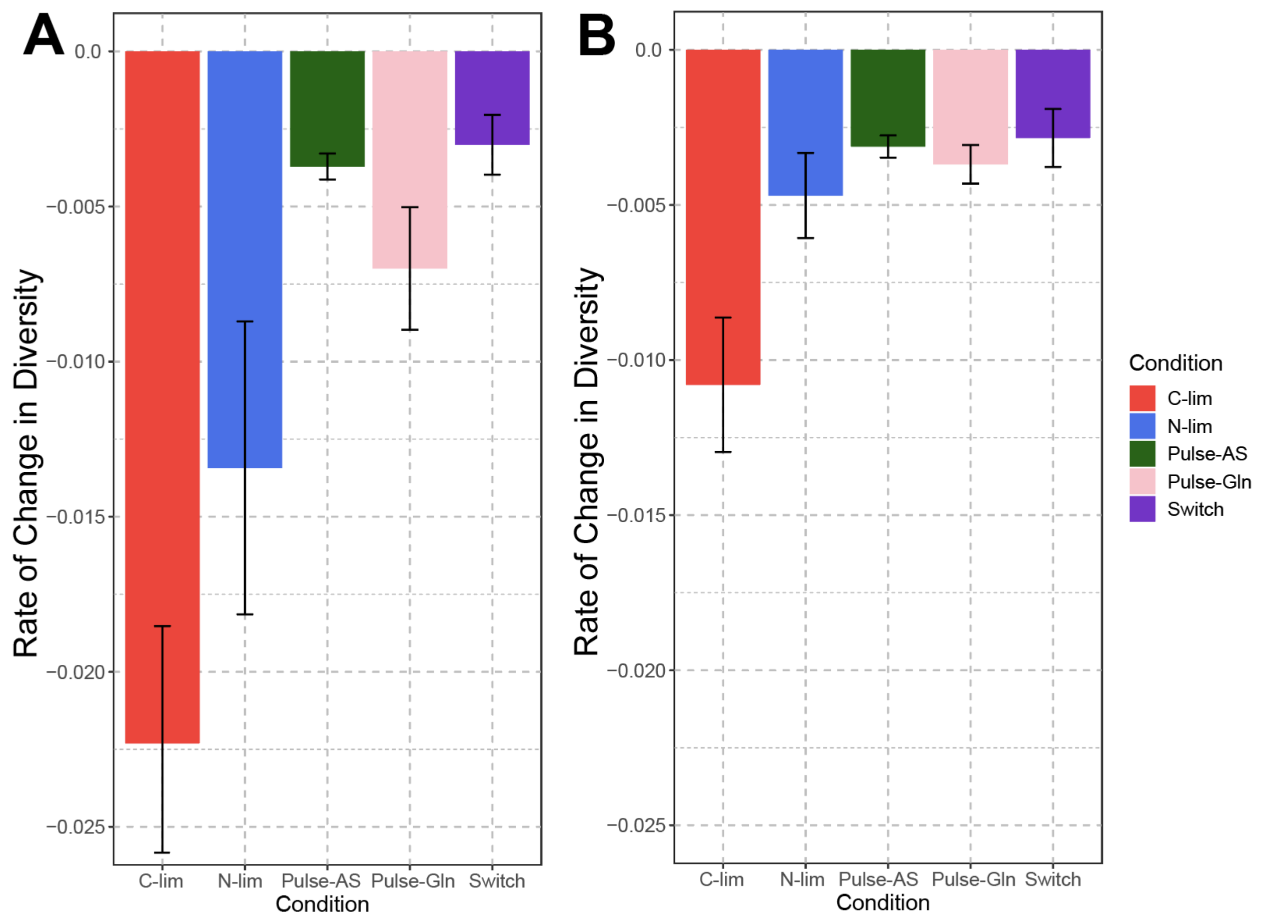

Supplemental figure 3. Maintenance of genetic diversity in different fluctuating selections. (A) Quantification of the dynamics of genetic diversity in other selective conditions. **(B)** Quantification of the dynamics of genetic diversity after excluding the highest fitness genotype in each condition. Error bars indicate the upper and lower bounds of the 95% confidence interval in each condition.

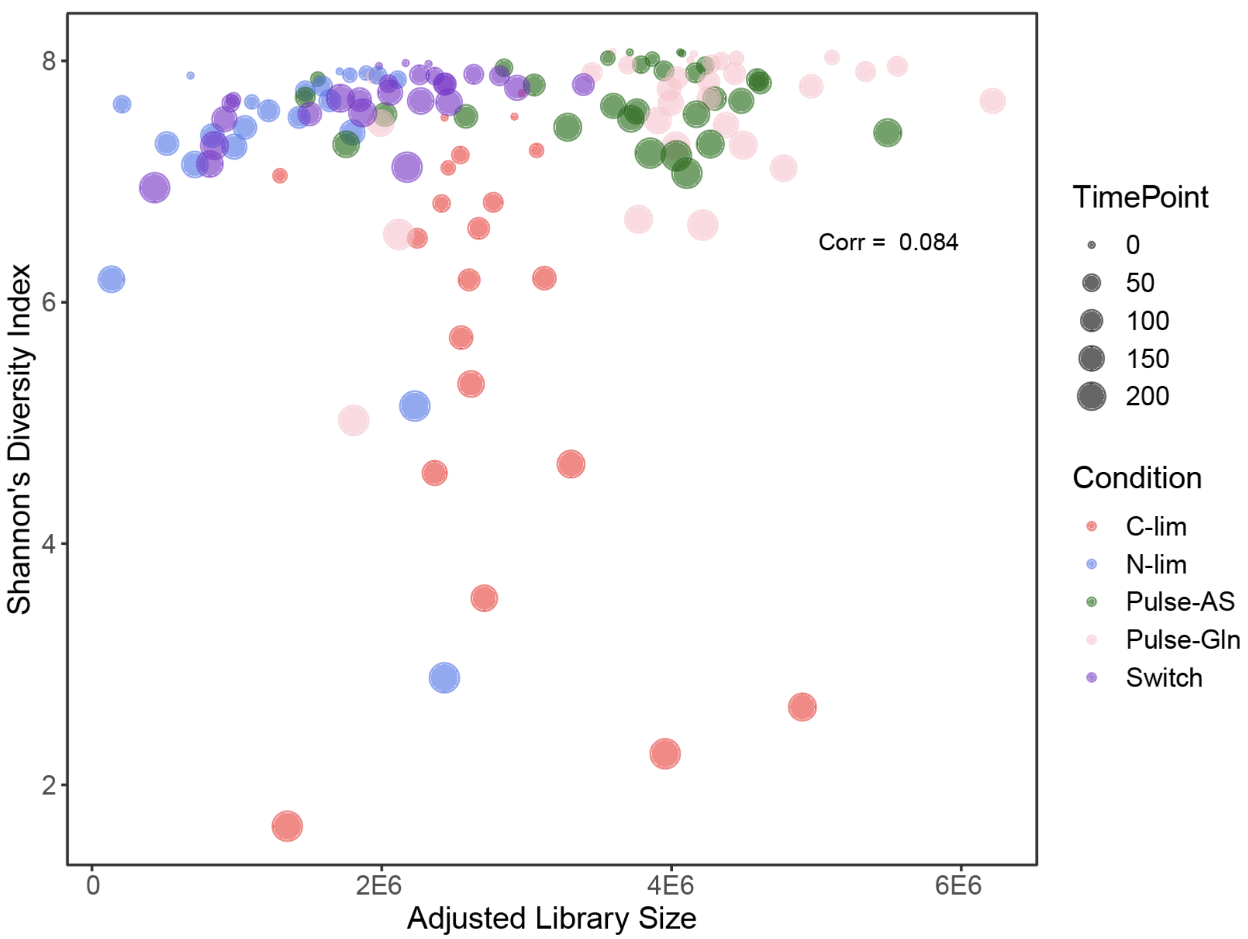

Supplemental figure 4. Library size does not affect diversity estimates. Plot showing the relationship between Shannon's diversity index and adjusted library size.

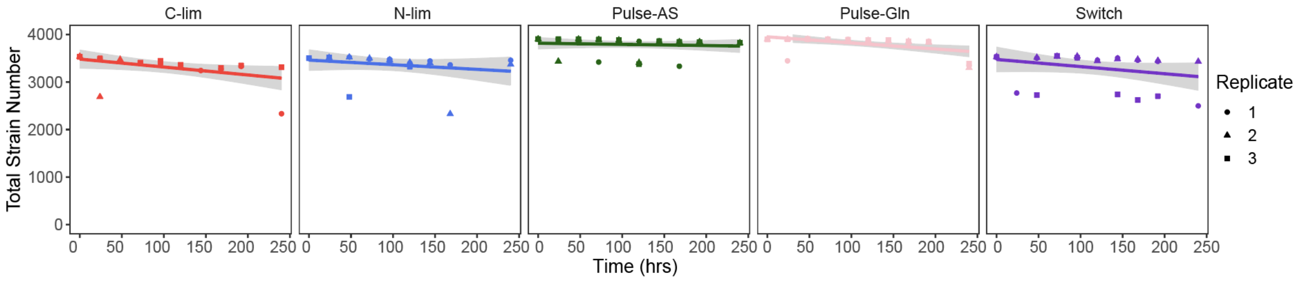

Supplemental figure 5. The rate of change in total strain number across all conditions.

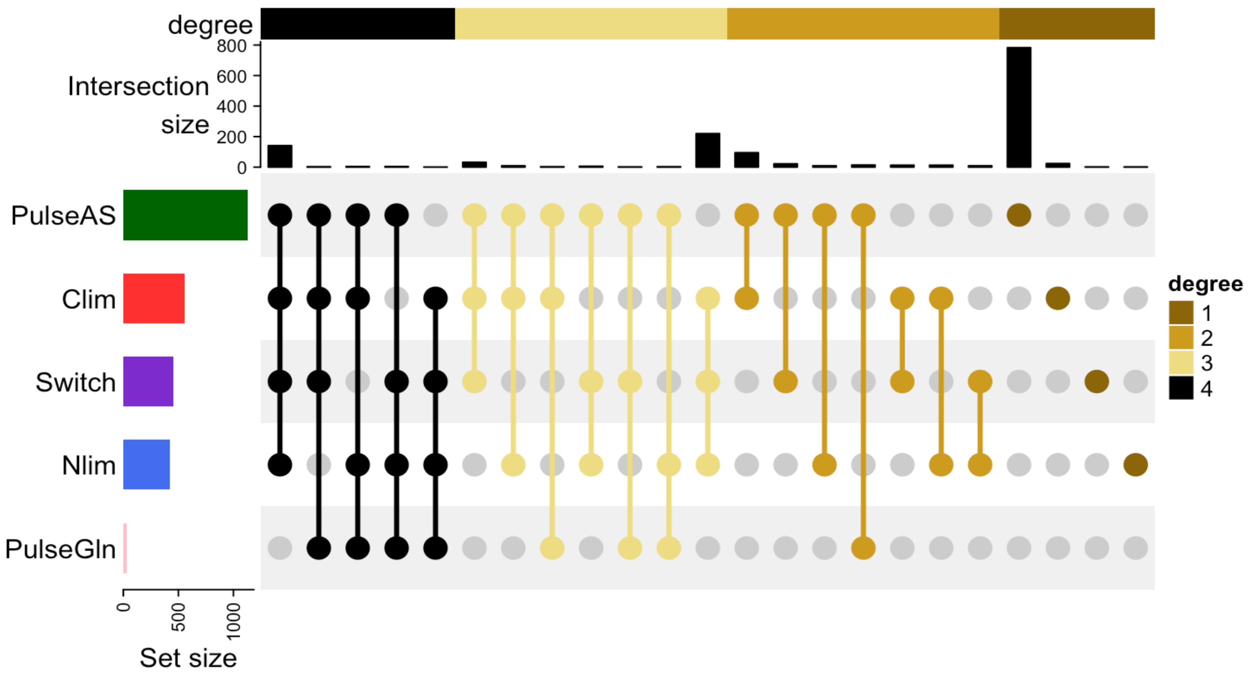

Supplemental figure 6. Strain extinction profiles in each condition. Extinct strains in the final time point (t = 240) are shared between subsets of conditions. Degree refers to the number of conditions that share a set of extinct genotypes.

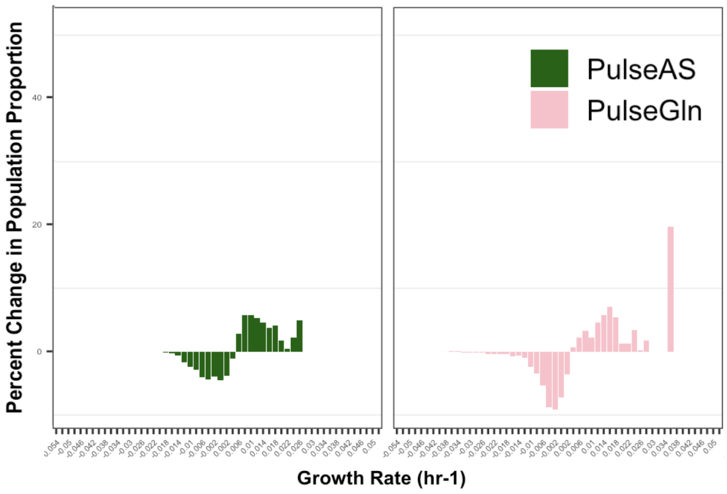

Supplemental figure 7. Change in percent population proportion for pulse conditions.

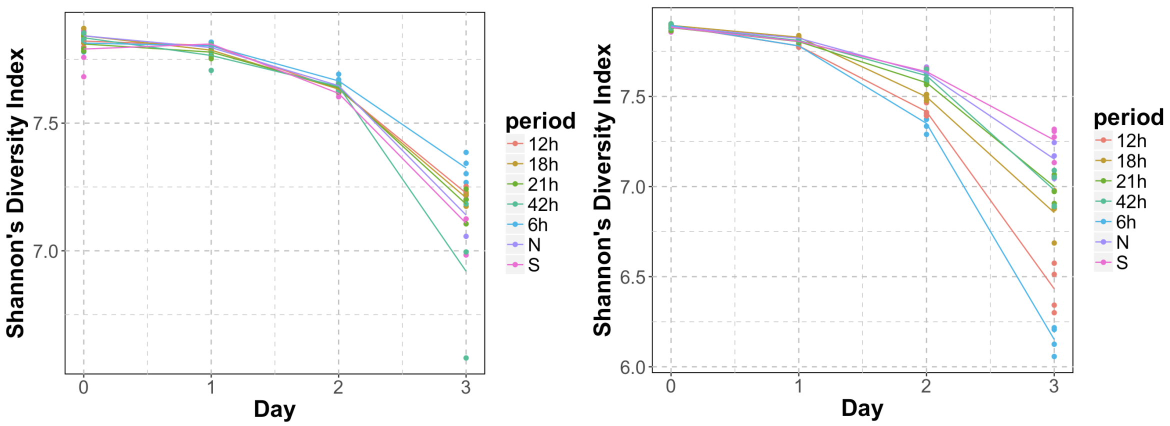

Supplemental figure 8. Diversity measurements of experiments from the Salingnon et al. dataset. Barseq was performed on the haploid gene deletion yeast library in conditions fluctuating between high (S) and low methionine (N) concentrations (left) and conditions fluctuating between salt (S) and no salt (N) concentrations (right).

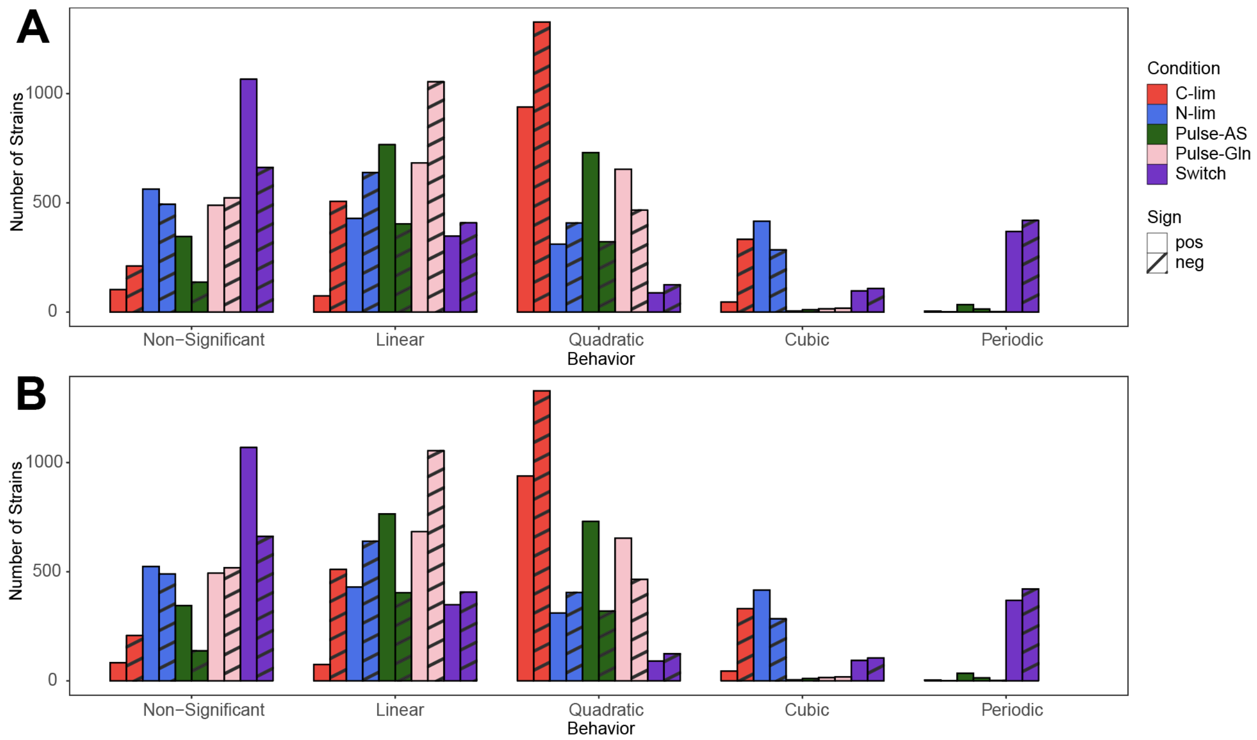

Supplemental figure 9. Genotype dynamics in static and fluctuating environments. (A) Extended summary of growth behavior including the two additional pulse conditions**. (B)** Reanalysis following removal of the highest fitness genotype does not alter the distribution of model fits**.**

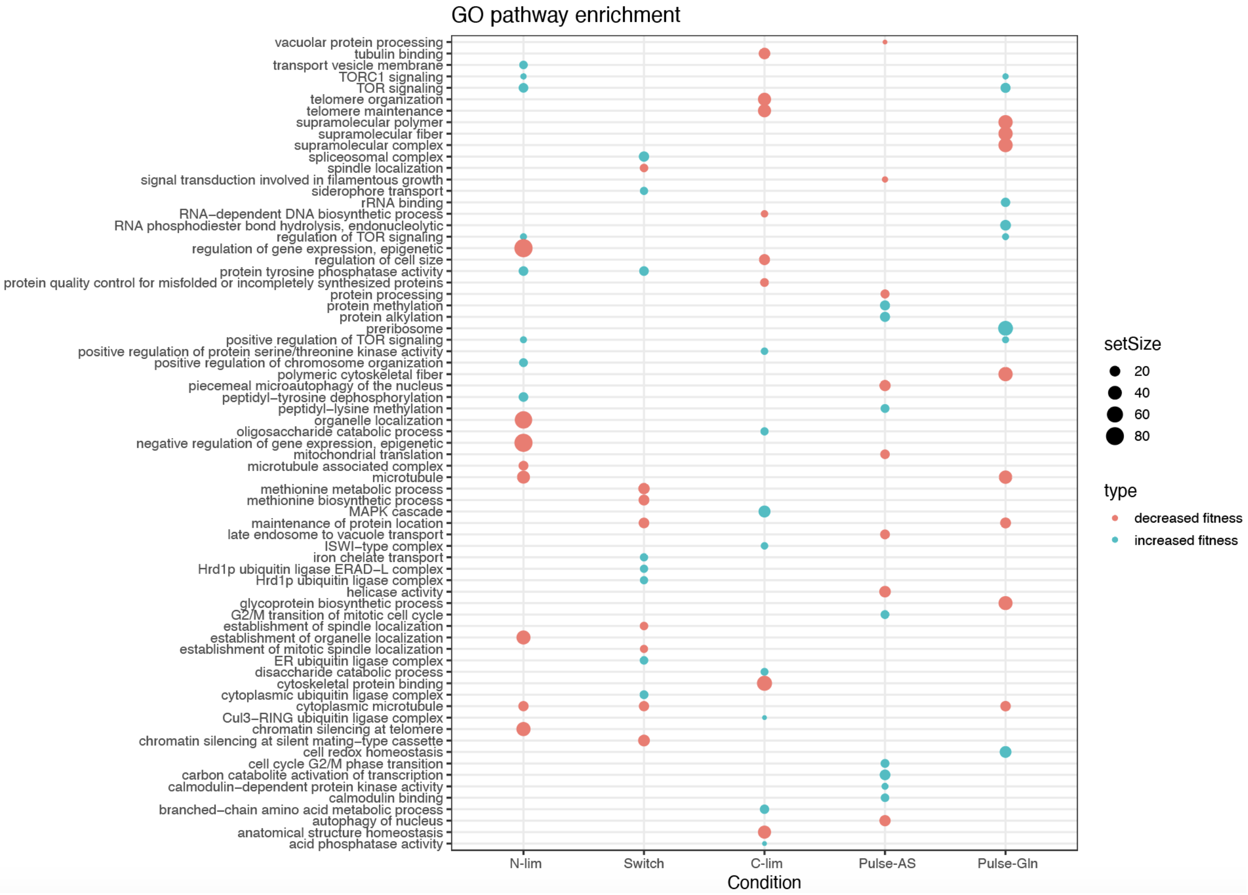

Supplemental figure 10. Gene set enrichment analysis (GSEA) of fitness effects in each condition. The top and bottom eight significantly (p-value < 0.05) represented GO terms for each condition are shown. Set size refers to the number of genes contained in a category.

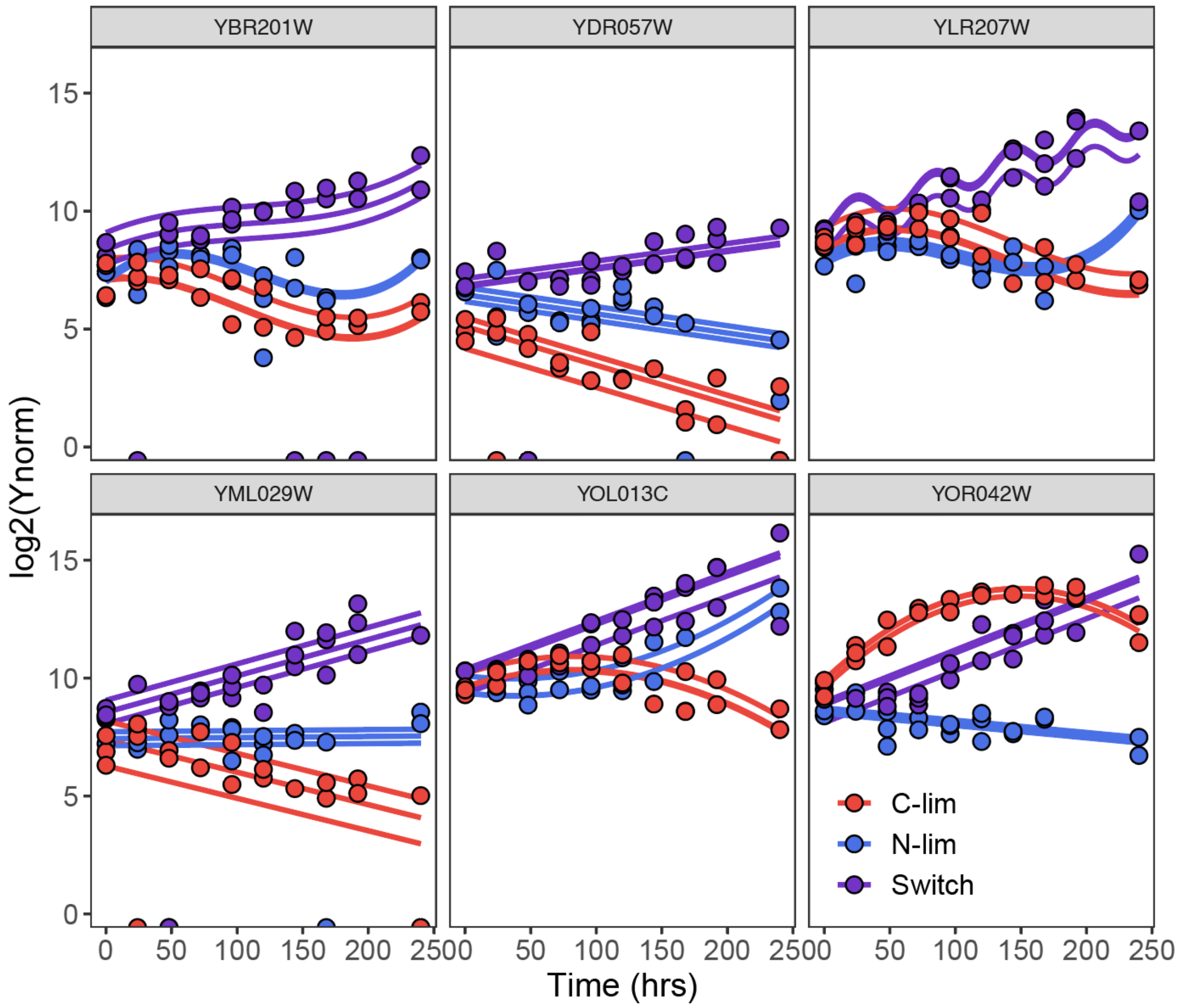

Supplemental figure 11. Deletion of the ERAD genes uniquely results in increased fitness in fluctuating environments. DER1, YOS9, HRD3, USA1, HRD1, and CUE5 gene deletions show consistent significant fitness increase in the switch condition but variable responses in carbon and nitrogen limiting conditions.

SUPPLEMENTARY Tables

Supplemental table 1. Pairwise correlation matrix of counts across all conditions.

Supplemental table 2. DFE statistical measurements

Condition	Mode	Median	Mean	Max	Min	Range	Variance
C-lim	0.0077	-0.0031	-0.0027	0.0478	-0.0271	0.0749	6.53E-05
N-lim	0.0124	-0.0003	-0.0007	0.0435	-0.0439	0.0874	8.64E-05
Switch	0.0047	0.0005	-0.0018	0.0265	-0.0504	0.0769	9.53E-05
Pulse-AS	0.0020	0.0031	0.0027	0.0278	-0.0176	0.0455	4.29E-05
Pulse-Gln	-0.0026	-0.0004	-0.0012	0.0361	-0.0415	0.0776	6.94E-05
